# Physiological responses and reproductive performance of naturally heat-stressed rabbit does treated with postbiotic of *Bacillus subtilis* and *Saccharomyces cerevisiae* in free and nano-encapsulated forms

**DOI:** 10.1186/s12917-025-04728-6

**Published:** 2025-04-25

**Authors:** N. S. Hosny, A. S. Morsy, Z. R. Abo-elezz, N. M. Hashem

**Affiliations:** 1 Livestock Research Department, Arid Lands Cultivation Research Institute, City of Scientific Research and Technological Application (SRTA-CITY), New Borg El Arab, Alexandria, Egypt; 2 Department of Animal and Fish Production, Faculty of Agriculture, Alexandria University, Alexandria, 21545 Egypt

**Keywords:** Nano-encapsulation, Palm date by-product, Postbiotic, Fertility, Antioxidant, Heat stress

## Abstract

**Background:**

Heat stress negatively affects the physiology and reproductive functions of rabbits. In order to mitigate these effects, palm date seeds were fermented with *Bacillus (B.) subtilis* and/or *Saccharomyces* (*S.) cerevisiae* to generate microbial-derived active metabolites, postbiotics, which were subsequently characterized using GC-MS. Notably, the postbiotic generated by the combined microbial fermentation (BYP) exhibited the highest concentration of secondary active metabolites. This postbiotic was then incorporated into rabbit diets in either free (BYP) or nano-encapsulated (NBYP) formulations.

**Methods:**

Forty-five nulliparous rabbit does were randomly assigned to one of three groups and received dietary supplementation with either 0.9 g/kg BYP, 0.9 g/kg NBYP, or no supplementation (C) over 30 days spanning mating and the first and second semesters of pregnancy.

**Results:**

Supplementation with BYP and NBYP significantly increased feed intake compared to the C group, while concurrently reducing rectal temperatures and respiratory rates. Both treatments markedly enhanced hematological, immunological, and redox parameters, as well as progesterone levels during pregnancy. The NBYP demonstrated superior effects for most variables, particularly during pregnancy. Additionally, the kindling rate and litter size and litter weight at birth were significantly higher in the BYN and NBPY groups compared to the C group.

**Conclusions:**

The incorporation of NBYP is recommended as an innovative natural microbial-derived supplement to enhance the health status, heat tolerance, and reproductive efficiency of rabbit does maintained under natural thermal stress conditions.

## Background

The raising of global warming poses a significant threat to livestock production, particularly in rabbit farming, due to the high susceptibility of rabbits to heat stress [1]. The optimal ambient temperature range for rabbits is 15–25 °C, whereas temperatures above 30 °C cause heat stress, resulting in significant changes in physiology, behavior, and metabolism. In adult rabbit does, heat stress correlates with reduced fertility and estrogen and progesterone secretion, impaired ovum integrity, and lower fertilization and conception rates, ultimately affecting litter size and litter weight [2–4].

Dietary manipulation is an effective strategy for alleviating the effects of heat stress in rabbit farming. Targeted supplementation may improve redox status, regulate inflammatory and immune responses, stabilize hormonal balance, and enhance overall reproductive performance. Microbial-derived supplements, including probiotics and postbiotics have been extensively studied for their health-promoting properties. Probiotics are acknowledged for their advantages. However, concerns persist regarding their viability, the possible presence of antibiotic-resistant genes, and their sensitivity to gastrointestinal and storage conditions, which may compromise their efficacy [5]. Furthermore, the sensitivity of probiotics to gastrointestinal conditions, industrial processing, and storage can limit their biological activity and restrict their utility as feed supplements [6, 7]. The concept of a “postbiotic” has emerged as an alternative in order to extend the scope of the probiotics concept beyond its inherent viability. The term “postbiotic” denotes secondary metabolites obtained from probiotic strains, such as *Lactobacillus (L.)*, *Bifidobacterium*, *Streptococcus,* and *S. cerevisiae* yeast [6, 7]. In 2019, the International Scientific Association of Probiotics and Prebiotics (ISAPP) broadened the conceptual framework of the term ‘postbiotic’ to encompass “the production of microorganisms that are non-living and/or their components that confer health benefits to the host organism.” Postbiotics provide an advantage over probiotics as their benefits do not rely on cell viability, which can vary, and the presence of dead cells may even exceed that of viable cells. The health benefits of postbiotics stem from multiple mechanisms, including antimicrobial, antioxidant, and immunomodulatory activities, along with improving gut function and preserving eubiosis. The observed effects are primarily due to postbiotic compounds such as bacteriocins, organic acids, enzymes, and vitamins [8, 9].

Although few studies have evaluated the health benefits of postbiotics in farm animals, current research suggests potential advantages. For instance, in heat-stressed broiler chickens, the addition of postbiotics derived from *L. plantarum* improved growth performance, intestinal morphology, immune response, the mRNA expression levels of hepatic insulin-like growth factor- 1, plasma immunoglobulins, and lactic acid bacteria count while reducing the *Enterobacteriaceae* count [10]. Another study demonstrated that the administration of postbiotic-stabilized non-viable *Lactobacilli* fermentation products in both dry and aqueous formulations ameliorated colisepticemia [11]. Postbiotics produced by fermentation of *S. cerevisiae* in transitional dairy cows improved milk yield, antioxidant capacity, and immunity [12]. Additionally, lactic acid bacteria-based postbiotics enhanced sperm quality and overall health in male rabbits [13].

Postbiotic quality and its enrichment with secondary metabolites mainly depend on the microbial strains used and the composition of the fermentation media, which influence the metabolic output of these strains. For instance, the addition of inulin to the fermentation medium enhanced the inhibitory activity of *L. plantarum* postbiotics against various pathogenic bacteria, including *S. enterica, E-coli, and L. monocytogenes* [14]. Consequently, the product enhanced the broiler chickens'fecal microbiota, meat quality, growth performance, and growth-linked gene expressions [15]. Chang et al. [16] found that the media type affected the composition and functional characteristics of postbiotics generated by *L. plantarum*, and specific metabolites, particularly pyrrole compounds, can be generated by regulating fermentation media composition.

Based on previous findings, we aimed to develop a postbiotic enriched with diverse secondary metabolites to mitigate the adverse effects of heat stress on rabbit does during mating and pregnancy while ensuring product safety by eliminating synthetic media in the production of microbial secondary metabolites. Therefore, we utilized two well-established safe and effective probiotics, *B. subtilis and S. cerevisiae* [17, 18]. Moreover, we used palm date (*Phoenix dactylifera L. Arecaceae*) seeds by-product as a fermentation medium due to their cost-effectiveness, particularly in arid and semi-arid regions, as well as their high nutritional value and enrichment with nutrients and vitamins that facilitate microbial growth [19]. Additionally, we evaluated the efficacy of free versus nano-encapsulated postbiotics to determine whether nano-encapsulation enhances the biological activity of the secondary metabolites, as recommended by previous studies [20, 21].

## Methods

### Study location

The fieldwork was conducted at the Rabbit Physiology Research Laboratory, Agricultural Experimental Station, Faculty of Agriculture, Alexandria University, Egypt. The postbiotics preparation and laboratory analyses were performed at the Nanoencapsulation and Biotechnology Laboratories (NBL, Animal and Fish Production Department, Faculty of Agriculture, Alexandria University) and at the Laboratory of Livestock Research (Arid Land Cultivation Research Institute, City of Scientific Research and Technological Applications, Alexandria). All procedures were approved by the Committee for Institutional Animal Care and Use at Alexandria University (Approval No. 082211212118).

### Postbiotic preparation

Dates (*Phoenix dactylifera*) were obtained from a palm farm in El Kharga Oasis, The New Valley, South Egypt (25°26'18''N, 30°33'30''E). The date seeds were manually separated from the flesh, thoroughly washed, and stripped of excess husk. The seeds were then dehydrated at 50 °C for 2 days and finely milled to a particle size of 1.0 mm using a heavy industrial disc mill (Buhler-Miag laboratory disc mill, model DLFU, Shandong Province, China).

Probiotic strains *B. subtilis* (EMCC 1009, isolated from natto food, Japan) and *S. cerevisiae* (ATCC MYA- 795, isolated from cream) were obtained from Cairo MIRCEN (Faculty of Agriculture, Ain Shams University, Cairo, Egypt).

*B. subtilis* was activated on DSM agar plates (composition: peptone, 5 g; meat extract, 5 g; agar, 15 g per liter; pH 7.0) to prepare activated colonies for subsequent inoculation. Nutrient broth (composition: D(+)-glucose, 1 g; peptone, 15 g; sodium chloride, 6 g; and yeast extract, 3 g per liter; pH 7.5) was used for *S. cerevisiae* activation. Both media were prepared in 500 mL flasks, sterilized by autoclaving at 121 °C for 15 minutes, and cooled before inoculation [1]. Each microbial strain was cultured on agar plates at 30 °C for 48 hours.

To produce postbiotics, date seeds powder (1 kg) was soaked in distilled water (1 L) and inoculated with either *B. subtilis* (8 × 10^11^ CFU), *S. cerevisiae* (8 × 10^11^ CFU), or both strains combined (8 × 10^11^ CFU of each). The mixtures were incubated at room temperature for three days [22]. Following fermentation, the contents were centrifuged (Benchtop Microfuge 20 R, Beckman Coulter, Germany) at 10,000 g for 15 minutes at 4 °C. The resulting cell-free supernatants (postbiotics) were collected, lyophilized, and stored at − 80 °C for further analysis.

### Identification of the active components of postbiotics

The chemical constituents of non-fermented date seeds extract and *B. subtilis*, *S. cerevisiae*, and *B.subtilis and S. cerevisiae* postbiotics were characterized using gas chromatography coupled with mass spectrometry (GC-MS; Thermo Scientific TRACE- 1300 series GC; Thermo Fisher Scientific Inc., Austin, TX, USA). The analysis was performed on a fused silica DB- 5 capillary column (30 m length, 0.32 mm inner diameter, 0.25 µm film thickness; Thermo Fisher Scientific Inc., TSQ 8000 Evo). The temperature profile for the column oven was initially held at 50 °C for 2 minutes, subsequently increased at a rate of 5 °C/min to reach 250 °C for an additional 2 minutes, and finally elevated to 300 °C at a rate of 30 °C/min for 2 minutes. Helium was employed as the carrier gas, maintaining a continuous flow rate of 1 mL/min. The injector and detector temperatures were set at 250 °C and 290 °C, respectively. Mass spectra were recorded at 5 scans per second over a mass-to-charge ratio of 40 to 700 amu. The NIST 14 mass spectral database, incorporating retention index data, was used for the identification of the chemical constituents [23]. Based on the GC-MS findings, postbiotic date seeds fermenterd with bothd *B. subtilis* and *S. cerevisiae* (BYP) was selected for further investigation.

### Creation and characterization of the nano-encapsulated postbiotic

As per the ionic gelation procedure, sodium alginate and calcium chloride (CaCl_2_) were utilized to synthesize NBYP [23]. First, a 1% (w/v) sodium alginate solution was mixed with 0.9 g of BYP under continuous gentle magnetic stirring. Subsequently, two portions of the mixture were injected dropwise using a syringe pump (Model: SK 500I, Shenzhen, China) into one portion of CaCl₂ solution (2.2 mol/L). The resultant nanoparticles were collected by centrifugation at 8,000 rpm for 20 minutes and stored at − 80 °C. A scientific nanoparticle analyzer (Zetasizer Nano ZS, Malvern Instruments Ltd., Worcestershire, UK) was used at 25 °C to assess the physicochemical properties, including particle size, polydispersity index (PdI), and zeta potential of both the sodium alginate-CaCl₂ complex and the NBYP.

### Animals and experimental design

A total of 45 nulliparous rabbit does, aged 5 months and weighing 2.920 ± 0.10 kg, were used. The does were individually housed in standard wire cages under same management conditions [24]. Rabbit does were fed a pelleted diet containing 28% alfa alfa hay, 18% barley, 25% wheat bran, 6% yellow corn, 18% soybean, 2% molasses, 1% di-calcium phosphate, and 1% NaCl, and 1% premix, covering daily maintenance as recommended by the National Research [25]. The chemical analysis of the diet (g/100 g DM) was: 17.50 crude protein, 2.05 ether extract, 2.53 crude fiber, 9.43 ash, and 59.45 nitrogen-free extract. Rabbit does were divided into three homogenous experimental groups and received 900 mg/kg of diet free postbiotic (BYP), 900 mg/kg of diet nano-encapsulated postbiotic (NBYP), or no supplementation (control, C). Treatments were administered for 30 days, including 10 days pre-mating and 20 days post-mating (first and second semesters of pregnancy). Each rabbit doe received 25 IU of equine chorionic gonadotropin (Gonaser®, Hipra, Spain) intramuscularly to achieve estrous synchronization. Forty-eight hours later, 0.8 µg of gonadotropin-releasing hormone (0.8 µg buserelin; Receptal, Boxmeer, Holland) was administered intramuscularly to induce ovulation. This was followed by artificial insemination of 0.2 mL of freshly diluted (1:5) pooled semen containing 15 × 10⁶ sperm/insemination [21].

### Metrological variables and heat-tolerance indices

An electronic digital thermo-hygrometer was used to record the rabbitry's ambient temperature (ºC) and relative humidity during the experimental period. The formula db ºC- [(0.31 - 0.31 RH%) (db ºC- 14.4)] was used to calculate the temperature-humidity index (THI), where RH% represents the percentage of relative humidity, while db℃ denotes the dry bulb temperature measured in degrees Celsius. The THI values were classified as follows: the absence of heat stress (27.8), moderate heat stress (27.8–28.8), severe heat stress (28.9–29.9), and extremely severe heat stress (> 30.0) for rabbits [26]. The mean values for ambient temperature, relative humidity, THI, and day length were 30.30 ± 0.12 ºC, 72.40 ± 0.52%, 28.91 ± 0.113, and 15.08 ± 0.12 h, respectively. Rectal temperature, respiration rate, and feed intake of rabbit does were monitored and recorded to assess their heat-tolerance capacity [23].

### Hematobiochemical attributes

Blood samples were collected from the marginal ear veins on the day of insemination (day 0) and on days 10 and 20 post-mating (first and second semesters of pregnancy) using heparinized vacuum tubes (blood collection vacuum tubes, REF: G40111, NEW VAC, China). Each sample was divided into two subplots to assess immunological variables and biochemical characteristics: whole blood and separated blood plasma. Blood plasma samples were obtained by centrifugation at 3,000 g for 20 min at 4℃ [27]. Red blood cells (RBCs), white blood cells (WBCs) and their types, and hemoglobin levels were assessed according to El-Desoky et al. [23]. Blood plasma samples were analyzed for total protein, albumin, and glucose concentrations using a BioSystem SA kits (Barcelona, Spain), with method linearity ranges of ≤ 10.0 g/dL, ≤ 7.0 g/dL, and ≤ 500 mg/dL, respectively. Globulin concentrations were calculated by subtracting albumin values from total protein values. Furthermore, the antioxidant capacity of blood plasma was evaluated using colorimetric commercial kits (Biodiagnostic, Giza, Egypt) by measuring the activity of reduced glutathione enzyme (GSH-Px), malondialdehyde (MDA, linearity of the methods was up to 100 nmol/mL), and total antioxidant capacity (TAC, linearity of the methods was up to 2 mM/L). The plasma lysozyme activity (LA) analysis was conducted following the methodology outlined by Hashem et al. [28]. The interlukin- 1β ELISA (IL- 1β) kit (Cat. No. MBS262525, My BioSource, San Diego, CA 92195–3308, USA) was utilized to measure IL- 1β levels in blood plasma, with sensitivity limit of 5 pg/mL and intra- and inter-assay precisions of ≥ 8% and ≥ 12%, respectively [24]. Insulin-like growth factor l (IGF-l) concentrations were assessed using an immunoassay kit (Quantikine IGF-l Immunoassay, R&D Systems, Minneapolis, MN, USA). An enzyme-linked immunosorbent assay (IBL America Immuno-Biological Laboratories, Inc., Minneapolis, MN, USA) was used to measure the amounts of immunoglobulin G (IgG), -A (IgA), and -M (IgM). The assay demonstrated a sensitivity of 90.0% (95% confidence interval: 68.3% - 98.7%) for IgM and IgG and 100% (95.0% confidence interval: 95.2% - 100%) for IgA.

Progesterone concentrations in blood plasma samples collected during pregnancy (days 10 and 20) were analyzed using commercial solid-phase enzyme immuno-assay ELISA kits purchased from Pointe Scientific Inc, MI, USA. The analysis demonstrated a sensitivity of 0.0625 ng/mL. The corresponding intra-assay and inter-assay coefficients of variation were 2.4% and 2.6%, respectively.

### Reproductive performance

Reproductive and pregnancy variables, including litter size at birth (total of kits, both viable and non-viable rabbits), weight of the litter at birth, and kindling rate ([number of delivered females/number of inseminated females × 100]) were measured [23].

### Statistical analyses

All statistical analyses were conducted using the Statistical Analysis Software program (SAS, Version 8, Cary, NC, USA: SAS Institute; 2001). The fixed effects of treatment (C, BYP, and NBYP), status (physiological status at the time of sampling and/or data collection), and the treatment by status interaction were assessed for physiological and hematochemical variables, redox status, and hormonal profiles using the MIXED procedure for repeated measurement. The effects of treatments on litter size, litter viability, and litter weight were evaluated using one-way ANOVA, while the effect of treatments on the kindling rate was evaluated using the Chi-square test. Differences between treatment means were evaluated using Duncan's multiple range.

## Results

### GC-MS and physicochemical analyses

The analysis of non-fermented date seeds extract revealed eight secondary metabolites, with oleic acid (39.10%) and 9,19-cyclolanost- 23-ene 3β,25-diol (22.11%) identified as the predominant compounds (Table 1). Postbiotic derived from *B. subtilis*-fermented date seeds contained ten compounds, with glycerin (63.12%) and psi,psi-carotene- 1,1',2,2'-tetrahydro- 1,1'-dimethoxy (9.58%) being the most abundant. The postbiotic derived from S. cerevisiae-fermented date seeds produced eight compounds, including glycerin (36.06%), d-lyxo-d-manno-nononic- 1,4-lactone (20.30%), and d-glycero-l-gluco-heptose (12.30%). Co-fermentation involving both microbial strains yielded ten bioactive compounds postbiotic, with (Z)− 18-octadec- 9-enolide (19.70%), torilinolein (18.52%), desulphosinigrin (17.76%), and oleic acid (10.64%) identified as the predominant components (Table 1).

**Table 1:** GC-MS analysis of active metabolites of date seeds extract and postbiotics of *Bacillus subtilis*, *S. cerevisiae,* and both microorganisms cultured in date seeds

**Compounds**	**Area, %**
**Date seeds extract**	
Oleic acid	39.10
9,19-cyclolanost- 23-ene 3 β,25 diol	22.11
1,3,5-triazine- 2,4,6 -triamine	11.17
Methyl palmitate	10.56
Stigmast- 5-en- 3-yl 9-octadecenoate	8.52
9,19-cyclolanost- 24-en- 3-ol,(3.β)	7.00
Docosane	0.84
2-methyl- 6-propyl dodecane	0.70
***Bacillus subtilis* postbiotic**	
Glycerin	63.12
psi.,.psi.-Carotene,1,1',2,2'-tetrahydro- 1,1'dimethoxy	9.58
l-Gala-l-ido-octonic	4.60
d-Gala-l-ido-octonic amide	3.50
2,4-Difluorobenzene, 1-benzyloxy-	3.27
Benzaldehyde,3-benzyloxy − 2-fluoro- 4-methoxy	3.27
10-Phenyldecanoic acid	3.27
5-Benzyloxy- 3,8,9-trioxa-tricyclo[4.2.1.0(2,4)]nonane	3.27
á-D-Glucopyranose,4-O-á-D-galactopyranosyl	3.09
Lactone	3.01
***Saccharomyces cervisiae* postbiotic**	
Glycerin	36.06
d-Lyxo-d-manno-nononic- 1,4-lactone	20.30
d-Glycero-l-gluco-heptose 1-	12.30
Heptatriacotanol	11.68
d-Gala-l-ido-octonic amide	10.15
Trilinolein	4.65
Linolenic acid, 2-hydroxy- 1-(hydroxymethyl)ethyl ester (Z,Z,Z)-	3.69
2,4-Difluorobenzene, 1-benzyloxy-	1.17
***Bacillus subtilis and Saccharomyces cervisiae* Postbiotic**	
(Z)− 18-Octadec- 9-enolide	19.70
Trilinolein	18.52
Desulphosinigrin	17.76
Oleic Acid	10.64
1,1,3,3,5,5-Hexamethyl- 1,5-bis(2-me thylpropoxy)trisiloxane	9.76
[1,1'-Bicyclopropyl]− 2-octanoic acid, 2'-hexyl-, methyl ester	8.05
d-Mannose	5.39
Estra- 1,3,5(10)-trien- 17á-ol	4.81
Strychane, 1-acetyl- 20à-hydroxy- 16-methylene-	2.74
Cyclopropanebutanoic acid, 2-[[2-[[2-[(2 pentylcyclopropyl)methyl]cyclopropyl]methyl]cyclopropyl] methyl]-, methyl ester	2.63

The physicochemical assessment of alginate-CaCl_2_ nanoparticles and alginate-CaCl2 nano-encapsulated BYP revealed average particle sizes of 195.10 nm and 325.3 nm, zeta potential measurements of − 3.41 mV and 8.88 mV, and polydispersity index (PdI) values of 0.457 and 0.551, respectively (Table 2).

**Table 2:** Physicochemical characteristics of the alginate–calcium chloride nanoparticles (A) and prepared nano-encapsulated postbiotic (B)

Item	A	B
Size, nm	195.10	325.3
Zeta potential, mV	− 3.41	8.22
Poly dispersity index	0.457	0.551

### Heat-tolerance indices

**Fig. 1:**
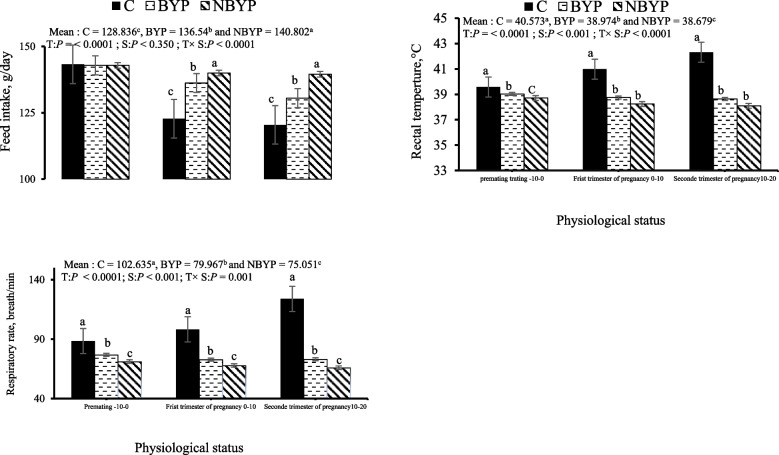
Changes (means ± SEM) in the feed intake, respiratory rate and rectal temperature of rabbit does during the experimental period. C = 0 mg/kg diet, BYP= 900 mg/kg diet, and NBYP = 900 mg/kg diet. Means within the same physiological status having different superscripts (a, b, c) differ significantly at *p* < 0.05

The effects of BYP (free postbiotic) and NBYP (nano-encapsulated postbiotic), at a dosage of 900 mg/kg diet on heat-tolerance indices, including feed intake, rectal temperature, and respiratory rate of rabbit does in various physiological conditions are shown in Fig. 1. Treatment with BYP or NBYP significantly increased the overall mean of feed intake. However, the interaction between treatment and physiological status indicated that this enhancement occurred during the first and second semesters of pregnancy (day 10–20 post-mating) but not during the pre-mating period. BYP and NBYP treatments significantly reduced rectal temperatures and respiratory rates in females across various physiological states (pre-mating, first and second trimesters of pregnancy) compared to the C treatment, with the lowest values recorded for NBYP treatment.

#### Hematobiochemical attributes

**Fig. 2:**
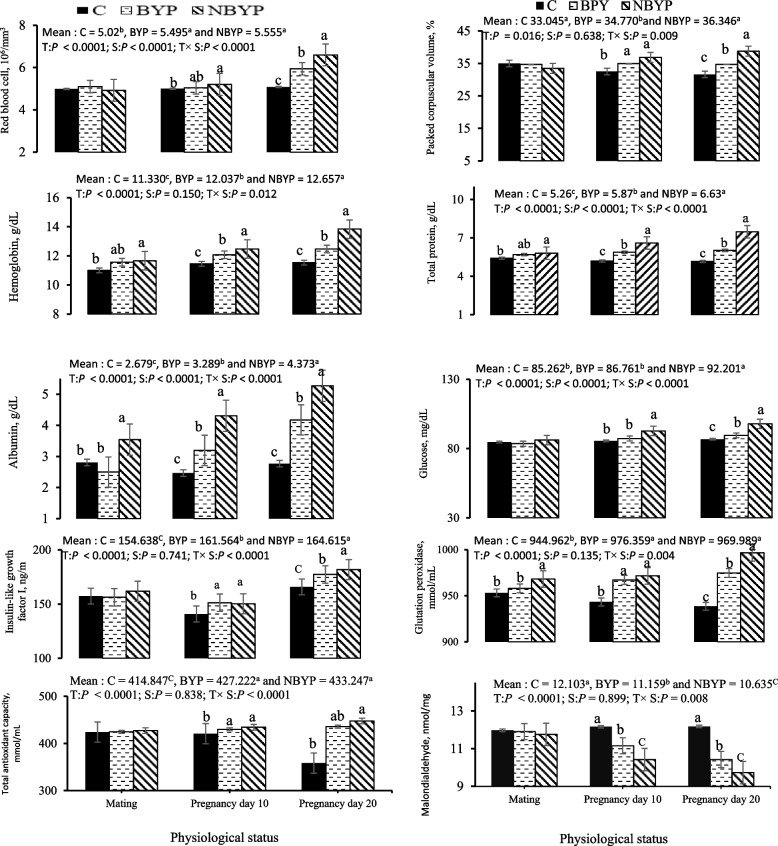
Changes (means ± SEM) in the blood plasma metabolites of rabbit does during the experimental period. C = 0 mg/kg diet, BYP= 900 mg/kg diet, and NBYP = 900 mg/kg diet. Means within the same physiological status having different superscripts (a, b, c) differ significantly at *p* < 0.05

The effects of BYP (free postbiotic) and NBYP (nano-encapsulated postbiotic), at the level of 900 mg/kg diet, on hematobiochemical attributes (RBCs, PCV, hemoglobin, total protein content, albumin content, glucose content, IGF-l, GSH-Px activity, TAC and MDA content) compared to control are shown in (Fig. 2). Both PBY-based treatments significantly increased overall count of RBCs (*p* < 0.0001), hemoglobin levels (*p* = 0.012), and PCV(*p* = 0.009) compared to the C treatment. These increases were observed during days 10 − 20 of pregnancy. The highest values were observed in the NBYP treatment on day 20 of pregnancy. Both BYP and NBYP treatments significantly increased overall concentrations of blood plasma metabolites (total protein, albumin, glucose, and insulin-like growth factor, *p* < 0.0001). These increases were noted between days 10 and 20 of pregnancy. The NBYP treatment exhibited the highest values on day 20 of pregnancy. Both BYP and NBYP treatments significantly increased the overall concentrations of blood plasma metabolites, including total protein, albumin, glucose, and IGF-l (*p* < 0.0001). These increases were noted in total protein and albumin across various physiological statuses, with the highest values recorded in the NPBY treatment on day 20 of pregnancy. During day 20 of pregnancy, blood plasma glucose concentrations and IGF levels increased. Both BYP and NBYP treatments significantly elevated overall concentrations of TAC (*p* < 0.0001) and GSH-Px (*p* = 0.004) while reducing MDA (*p* = 0.008) compared to the C treatment. These increases in TAC and GSH-Px decreases in MDA levels were noted between days 10 and 20 of pregnancy.

**Fig. 3:**
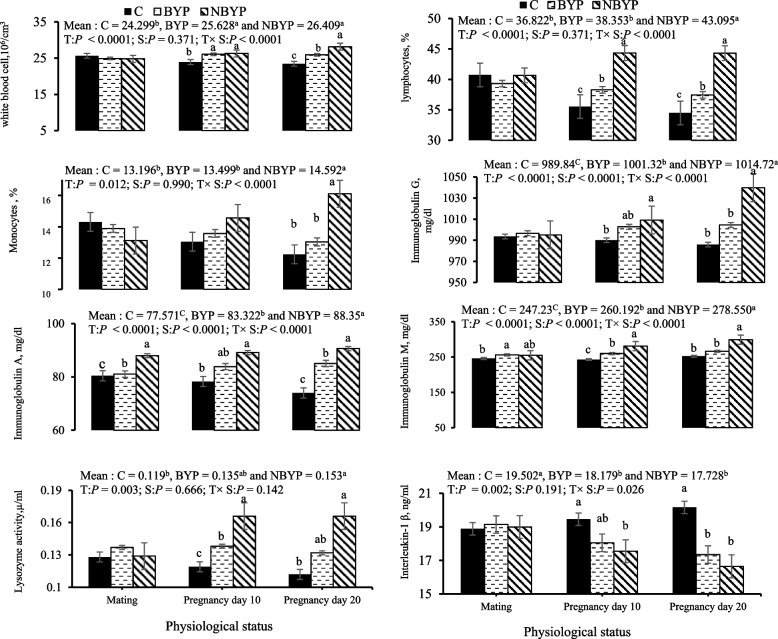
Changes (means ± SEM) in immunity variables of rabbit does during the experimental period. C = 0 mg/kg diet, BYP= 900 mg/kg diet, and NBYP = 900 mg/kg diet. Means within the same physiological status having different superscripts (a, b, c) differ significantly at *p <* 0.05

Both BYP and NBYP treatments significantly increased overall concentrations of WBCs count, lymphocytes, and monocytes (*p* = 0.05) compared to the C treatment (Fig. 3). Such increases were observed between days 10 and 20 of pregnancy for WBCs count and lymphocytes, while monocyte concentrations increased only on day 20 of pregnancy. Both BYP and NBYP treatments significantly elevated overall concentrations of blood plasma immunoglobulin G, M, and A (*p* < 0.0001) and lysozyme activity (*p* = 0.142), while reduced interleukin- 1β levels (*p* = 0.026) compared to the C treatment (Fig. 3).These increases were noted across various physiological statuses for IgA. During days 10 to 20 of pregnancy, an increase in IgG, IgM, and lysozyme activity was observed, with the highest values recorded in the NPBY treatment on day 20 of pregnancy. The concentrations of blood plasma interleukin- 1β significantly decreased between days 10 and 20 of pregnancy (Fig. 3).

#### Reproductive performance

**Fig. 4:**
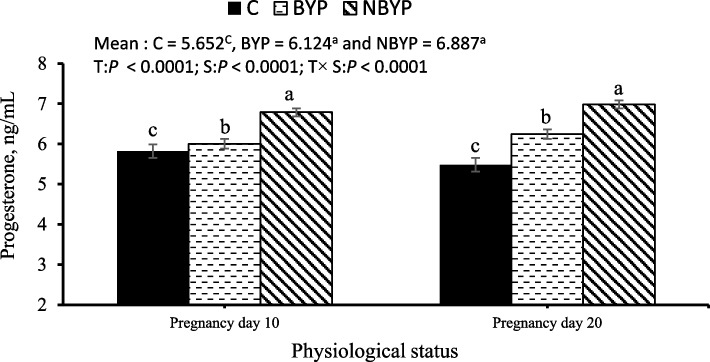
Changes (means ± SEM) in progesterone hormone of rabbit does during the experimental period. C = 0 mg/kg diet, BYP= 900 mg/kg diet, and NBYP = 900 mg/kg diet. Means within the same physiological status having different superscripts (a, b, c) differ significantly at *p* < 0.05

The effects of BYP (free postbiotic) and NBYP (nano-encapsulated postbiotic), at the level of 900 mg/kg diet, on reproductive performance indicators, including progesterone levels, kidding rate, litter size at birth, number of live and dead litter sizes, and litter weight at birth is presented in Fig. 4 and Table 3. Both BYP and NBYP treatments significantly increased overall concentrations of progesterone (*p* < 0.0001), with the highest concentration recorded in the NBYP treatment on day 20 of pregnancy (Fig. 4). Both BYP and NBYP treatments significantly enhanced kidding rates and live litter sizes, while reduced the number of dead litters compared to the C treatment (Table 3). NBYP demonstrated superior efficacy, yielding the highest values for all reproductive traits.

**Table 3:** Effect of treatments on the reproductive performance of rabbit does

**Variable**	**Treatment **^**1**^			**SEM**	***P-*Value**
	C	BYP	NBYP		
Kindling rate, %	53^c^ (8/15)	66^b^ (10/15)	80^a^ (12/15)	-	0.096
Litter size at birth	5.37	5.38	7.25	1.31	0.001
No. live litter sizes	3.87^b^	4.25^ab^	6.59^a^	1.48	0.03
No. dead litter sizes	1.51^b^	1.13^ab^	0.66^a^	0.92	0.04
Litter weight at birth, g	333.87^b^	401.63^b^	693.51^a^	74.6	0.001

^1^C = 0 mg/kg BW, BYP= 900 mg/kg BW and nano encapsulated NBYP = 900 mg/kg diet

Means within the raw having different superscripts (a, b, c) differ significantly at *p* < 0.05

## Discussion

This study investigated the potential of a postbiotic formulation to ameliorate heat stress negative impacts on thermotolerance and reproductive performance in rabbits. To safely promote animal health, date palm (Phoenix dactylifera L.) seed by-products were employed as a fermentation substrate for the probiotic strains *B. subtilis* and/or *S. cerevisiae*. Gas chromatography-mass spectrometry (GC-MS) profiling revealed that fatty acids, such as oleic acid, are the predominant secondary metabolites in the non-fermented date seeds extract. In contrast, postbiotics obtained from the fermentation of *B. subtilis* or *S. cerevisiae* postbiotics exhibited a higher concentration of carbohydrates, especially glycerol. Co-fermentation with both microbial strains produced novel secondary metabolites, such as trilinolein and desulfosinigrin, which were not present in single-strain postbiotics or non-fermented extracts. These findings highlight a synergistic metabolic interaction between *B. subtilis* and *S. cerevisiae*, which enhances the diversity of bioactive compounds and their functional effectiveness. Cross-strain interactions in mixed microbial cultures enable the integration of complementary biosynthetic pathways, optimizing the production of biologically active metabolites with improved host-targeted activity [29]. For example, Jia et al. [30] found that fattening lambs supplemented with *B.* *licheniformis* and *S. cerevisiae* combination had better growth performance, antioxidant capacity, immune function, ruminal fermentation, and microbial diversity than those supplemented with a single-strain.

Rabbit does subjected to severe heat stress (Temperature-Humidity Index = 28.91 ± 0.113) and supplemented with either BYP or NBYP demonstrated improved thermotolerance, as evidenced by increased feed intake, decreased rectal temperature, and respiratory rate, along with enhanced hematological and immunological functions, optimized blood metabolites, and better antioxidant status relative to the non-supplemented rabbit does (C). These findings are consistent with research indicating that *Aspergillus oryzae* postbiotics enhanceed heat stress resilience in cattle and Holstein calves [31]. In poultry, dietary supplementation with *L. plantarum* strains (RS5, RI11, UL4) attenuated heat stress-induced lipid peroxidation, restored antioxidant enzyme activity, and enhanced meat quality [32].

The hematobiochemical improvements observed in BYP/NBYP-supplemented rabbits, including elevated packed cell volume and hemoglobin levels are critical for sustaining thermoregulatory capacity under heat stress [33]. Both packed cell volume and hemoglobin levels improve oxygen transport and tissue delivery, facilitating cellular respiration and metabolic requirements during hyperthermia [23]. Albumin plays a crucial role in maintaining plasma colloid osmotic pressure, thereby preserving protein integrity and regulating fluid balance. Glucose serves as the main energy source in monogastric species, offering easily metabolizable energy and decreasing dependence on energy-demanding catabolic processes, particularly under high thermal conditions [23].

Heat stress adversely affects immune function, increasing infection susceptibility [3]. This study demonstrated that BYP and NBYP treatments significantly improved cellular immunity, as evidenced by increased lysozyme activity and decreased proinflammatory cytokines, as well as enhanced humoral immunity, indicated by elevated levels of immunoglobulins (IgG, IgA, IgM) relative to C treatment. These findings are consistent with the immunomodulatory effects of postbiotics documented in other studies. *Bifidobacterium* coagulans postbiotics induce T helper 2 cytokines (IL- 4, IL- 6, IL- 10), suppress IL- 2, and promote B-lymphocyte proliferation, exerting anti-inflammatory effects [34]. In another study, lactic acid bacteria postbiotic reduced tumor necrosis factor-alpha levels and increased interferon-gamma levels in vaccinated rabbits, thereby alleviating the severity of myxomatosis lesions in wild rabbits [35].

Interestingly, none of the previously published studies on postbiotics in farm animals have identified secondary metabolites of used postbiotics or their potential biological activities. In contrast to our study, the positive biological effects can be attributed to the distinct array of secondary metabolites found in BYP. For instance, trilinolein and desulphosinigrin exhibit antioxidant activity. Moreover, trilinolein is classified as a proinflammatory inhibitor of mediators for nitric oxide synthase, cyclooxygenase- 2, nuclear factor tumor necrosis factor- 1β, IL- 1, IL- 6, and mitogen-activated protein kinases [36]. Furthermore, oleic acid has antimicrobial activity. D-mannose plays a role in inhibiting bacterial adhesion [37] and is involved in the synthesis of glycoproteins, which are essential for immune system regulation and exhibit anti-inflammatory and antibacterial properties [38]. Additionally, [1,1′-bicyclopropyl]− 2-octanoic acid, 2′-hexyl-, methyl has anti-inflammatory, antitumor, antiviral, antibacterial, and antifungal properties [39].

The exposure of pregnant rabbit does to heat stress can evoke several reproductive hazards, including oocyte incompetence and decreased fertility, conception, and kindling rates, and increased embryonic loss and abortion rate [40, 41]. In this study, rabbit does supplemented with either BYP or NBYP expressed higher kindling rate, number of live litter size at birth, and litter weight at birth than the C rabbit does. This is primarily due to the decrease in proinflammatory cytokine levels, leading to increased uterine health and functioning [42]. Moreover, improved progesterone concentrations can contribute to these positive effects on conception rate and pregnancy outcomes [42]. Progesterone is responsible for the preparation of the myometrium for implantation by reducing muscle sensitivity to nervous or hormonal stimulation (oxytocin) and inhibiting lymphocyte proliferation and its activity [43]. Furthermore, several metabolites and hormones, notably glucose and insulin-like growth factor- 1, which showed significant improvement in the treated groups, can positively influence pregnancy development [43]. Additionally, the improvements in reproductive performance seen in this study may be directly attributable to the biological role of certain postbiotic metabolites. For example, oleic acids can enhance the development of one-cell rabbit embryos into morulae by serving as a reservoir for metabolic precursors found in uterine and oviductal fluids and embryos [44].

Finally, a key finding of this study is that NBYP exhibited greater biological activity than BYP, as indicated by the values for each variable, which were significantly higher or lower than those of BYP. These findings align with previous studies regarding the capacity of nanoparticles to traverse various biological barriers and sustain release over extended periods, thereby enhancing their cellular uptake and availability compared to larger-sized particles [20, 23].

## Conclusion

The secondary metabolites of postbiotics are affected by the type of the microorganism. This study demonstrates that the richest postbiotic, containing various secondary metabolites, was produced through the fermentation of date seeds using a combination of the microorganism strains *B. subtilis* and *S. cerevisiae*, rather than a single strain, thereby emphasizing the synergistic effects of both microbes. This postbiotic enhanced the health status and reproductive performance of rabbit does raise in natural heat conditions. Furthermore, the nano-encapsulation of the postbiotic improved its biological function, resulting in improved reproductive performance. Therefore, postbiotics represent a novel category of feed supplements that may effectively alleviate the effects of heat stress in rabbit does. Applying nano-encapsulation technology can yield further benefits. Future research should investigate alternative postbiotic formulations utilizing various fermenting materials and microorganisms for diverse applications in livestock production.

## Data Availability

Data is provided within the manuscript and any further information can be achieved by contacting the corresponding author.

## Abbreviations

BYP: Free postbiotic
C: Control
CaCl_2_: Calcium chloride
GC-MS: Gas chromatography/mass spectrometry
GSH-Px: Reduced glutathione enzyme
IgA: Immunoglobulin-A
IgG: Immunoglobulin-G
IGF-l: Insulin-like growth factor- l
IgM: Immunoglobulin-M
IL- 4: Interleukin- 4
IL- 6: Interleukin- 6
IL- 10: Interleukin- 10
LA: The plasma lysozyme activity
MDA: Malondialdehyde
NBYP: Nano-encapsulated postbiotic
P4: Progesterone
RBCs: Red blood cells
WBCs: White blood cells
